# Return-to-Play Post-Myocarditis for Athletes: To Play or Not to Play?

**DOI:** 10.3390/diagnostics14192236

**Published:** 2024-10-07

**Authors:** Kentaro Yamagata, Aneil Malhotra

**Affiliations:** Institute of Sport, Manchester Metropolitan University, Manchester M1 7EL, UK; k.yamagata@mmu.ac.uk

**Keywords:** myocarditis, athletes, cardiac magnetic resonance imaging, return-to-play

## Abstract

Myocarditis is a condition marked by inflammation of the heart muscle, which can lead to serious outcomes such as sudden cardiac death (SCD) and life-threatening arrhythmias. While myocarditis can affect any population, athletes, especially those engaged in high-intensity training, are at increased risk due to factors such as reduced immunity and increased exposure to pathogens. This review examines the clinical presentation, current guidelines, diagnostic challenges, and the significance of cardiac magnetic resonance imaging (CMR) in detecting myocardial inflammation and scarring. Current guidelines recommend a period of exercise restriction followed by thorough reassessment before athletes can return-to-play (RTP). However, there are several knowledge gaps, including the implications of persistent late gadolinium enhancement (LGE) on CMR and the optimal duration of exercise restriction. Additionally, the psychological impact of myocarditis on athletes highlights the importance of incorporating mental health support in the recovery process. A shared decision-making approach should be encouraged in RTP, considering the athlete’s overall health, personal preferences, and the potential risks of resuming competitive sports. We have proposed an algorithm for RTP in athletes following myocarditis, incorporating CMR. Future research is warranted to refine RTP protocols and improve risk stratification, particularly through longitudinal studies that examine recovery and outcomes in athletes.

## 1. Introduction

Myocarditis is an inflammatory process of the myocardium. This condition can present as an acute, subacute, or chronic disease process and may involve focal or diffuse myocardial tissue. While some patients with myocarditis may be asymptomatic, others may present with various symptoms, including chest pain, fatigue, dyspnoea, and congestive heart failure [1]. The severity of chest pain in myocarditis may vary, ranging from mild persistent chest pain observed in acute myopericarditis to symptoms resembling those of acute myocardial infarction [2,3]. The overall presentation of myocarditis can range from mild symptoms to severe cardiogenic shock, arrhythmias, and sudden cardiac death (SCD). Myocarditis is by far the most common acquired cause of SCD in young individuals [4,5].

Viral infections are the most common cause of myocarditis in developed countries, and it is estimated that up to 5% of all individuals with acute viral infections may have myocardial involvement. Although myocarditis can affect individuals of any age, it more frequently affects the young and previously healthy, with a male predominance. The incidence of myocarditis is estimated to be 10 to 20 cases per 100,000 persons, with approximately 1.5 million cases reported annually worldwide [1,3].

Myocarditis in athletes is an important disease entity. Although it rarely leads to significant morbidity and mortality in absolute terms, myocarditis remains one of the leading causes of SCD in competitive athletes [6,7,8,9,10]. Myocarditis may lead to life-threatening ventricular arrhythmias, which is why guidelines advise a comprehensive evaluation at baseline, exercise restriction, and re-evaluation before resuming competitive sport [11]. Adverse cardiac remodelling after myocarditis may lead to dilated cardiomyopathy (DCM) [12]. The pathways that govern this remodelling remain poorly understood.

A comprehensive assessment is essential to exclude atypical infections, systemic autoimmune diseases, and drug hypersensitivity as potential aetiologies. In addition, accurately identifying specific causes can guide the implementation of targeted therapeutic interventions [3]. Recommendations for clinical examinations and cardiac investigations, including advanced cardiac imaging for athletes with myocarditis before returning to play, were published by the European Society of Preventive Cardiology (EAPC), the European Society of Cardiology (ESC), and the American Heart Association (AHA)/American College of Cardiology (ACC) [11,13,14].

This review aims to address return-to-play (RTP) for athletes following myocarditis and the associated dilemmas.

## 2. Myocarditis in Athletes

While data on the general incidence of myocarditis in athletes are limited, a recent systematic review and meta-analysis found that COVID-19-related myocarditis, as detected by cardiac magnetic resonance imaging (CMR), ranged from 1% to 4% in approximately 8000 athletes who had recovered from COVID-19 [15]. Multiple registry data on SCD in athletes suggest that myocarditis accounts for 2% to 12% of all athlete fatalities. SCD due to myocarditis is thought to be due to cardiogenic shock or fatal arrhythmia [16].

**Figure 1:**
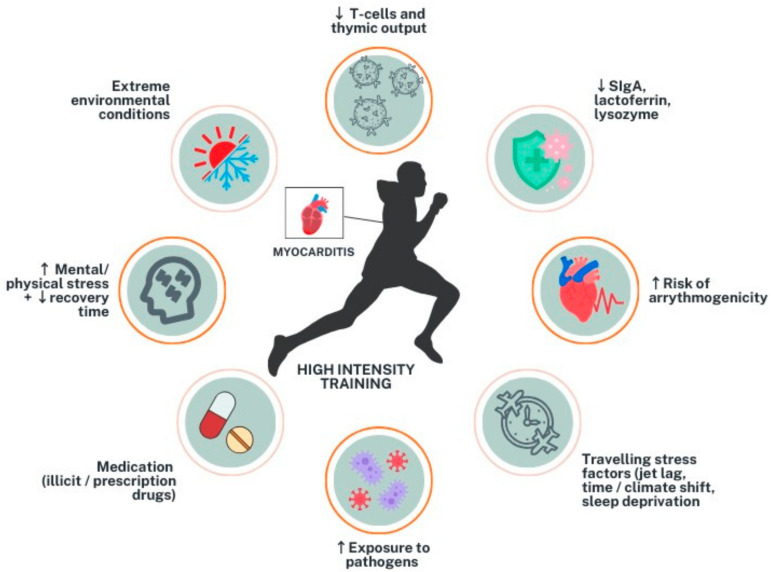
Factors contributing to increased risk of myocarditis in athletes. sIgA = secretory Immunoglobulin A; ↑ = increase; ↓ = decrease.

Compared to the general population, athletes may be at a higher risk of developing myocarditis, depending on the intensity of physical training [17]. While moderate-intensity training and exercise are typically considered cardioprotective, more intense training may increase the risk of viral upper respiratory tract infections by lowering immune response [17,18]. In fact, a J-curve relationship between exercise intensity and the risk of upper respiratory tract infection showed that moderate exercise decreases the risk of upper respiratory tract infections by 40–50%, whereas heavy exertion increases the risk by two- to six-fold [19,20]. Athletes who engage in high-intensity exercise are at a higher risk of developing myocarditis due to various factors (Figure 1). One of the main reasons is a decrease in innate immunity, including neutrophil respiratory bursts and natural killer cell activity, as well as a similar effect on the adaptive immune response. Studies have shown that elite athletes have severely reduced naïve T-cell numbers and thymic output, resulting in immune systems that mirror those of an older patient population [21,22]. Additionally, heavy exercise can reduce salivary secretory Immunoglobulin A, lactoferrin, and lysozyme, which can alter the T-cell response and lower immunity [23,24]. An augmented inflammatory response during exercise subjects athletes to a risk of atrial and ventricular arrhythmias and SCD [25,26]. Other contributing factors towards the higher prevalence of myocarditis in athletes include frequent travelling, exposure to pathogens, extreme environmental conditions, including excessive heat or cold and low humidity, competition-related stress, and medication use, including illicit drugs, doping agents, and other treatments such as antidepressants [27].

While the physical implications of myocarditis in athletes are well-documented, the psychological impact of such a serious cardiac diagnosis is equally significant. In a study by Asif et al., athletes diagnosed with serious cardiac conditions often experienced considerable psychological distress [28]. These athletes frequently avoid confronting emotions and discussions related to their diagnosis and should, therefore, be monitored and supported from a psychological perspective. It is thus important to incorporate psychological support as part of the comprehensive care plan for athletes with myocarditis to ensure that their mental well-being is addressed alongside their physical health. This approach may help prevent anxiety, depression, and other mental health issues that can arise due to the diagnosis and subsequent lifestyle changes.

## 3. Clinical Presentation and Diagnosis

Athletes with myocarditis present with heterogeneous symptoms, including chest pain, palpitations, dyspnoea, dizziness, and decreased exercise capacity [18,29]. Pyrexia and flu-like symptoms may also be present in cases of viral-induced myocarditis [30]. Symptoms may be nonspecific, and in some cases, diagnosis of myocarditis may be challenging. Moreover, diagnosis of myocarditis in elite athletes may be complicated by the presence of various electrocardiogram (ECG) changes commonly seen in this population, such as sinus bradycardia, early repolarisation, and T-wave changes in V2–V4 associated with a concave ST-elevation; the latter is considered to be a normal finding in black athletes [31].

The diagnosis of myocarditis involves a multifaceted approach that typically includes ECG, laboratory tests, echocardiography, CMR, and, in some cases, endomyocardial biopsy (EMB). ECG abnormalities present in approximately 85% of myocarditis cases and often include ST-segment elevations, particularly in the inferior and lateral leads, T-wave inversion, atrioventricular block, atrial fibrillation, tachycardia, and ventricular arrhythmias [29,30]. Laboratory diagnostics include myocardial necrosis markers such as high-sensitivity troponins and creatinine kinase-MB, with inflammatory markers such as elevated C-reactive protein and erythrocyte sedimentation rate also frequently observed. However, physicians need to keep in mind that cardiac markers such as troponin and creatine kinase may be increased after strenuous exercise activities in healthy athletes [32,33].

Echocardiography typically reveals a range of findings, from increased wall thickness and segmental hypokinesia to right ventricular dysfunction and pericardial effusion. Left ventricular ejection fraction (LVEF) at admission serves as an important prognostic indicator [30]. CMR is the gold standard for diagnosing myocardial inflammation due to its ability to detect changes independent of the underlying aetiology. CMR excels in multiparametric tissue characterisation, visualising the presence of oedema, hyperaemia, capillary leak, necrosis, and fibrosis, depending on the disease progression [34]. The updated Lake Louise Criteria integrate T2-weighted sequences for detecting myocardial oedema, T1-weighted images to assess non-ischaemic myocardial injury, as well as supportive findings including abnormal left ventricular (LV) systolic function and signs of pericardial inflammation, which are vital in assisting the diagnosis [35]. Late gadolinium enhancement (LGE) typically reveals non-ischaemic patterns in the sub-epicardial and mid-wall myocardium, with the lateral wall being a common site for LGE [36]. Although EMB is considered the reference standard for the diagnosis, it is not frequently performed due to its invasive nature and associated risks, with complications reported in up to 8.9% of patients [30]. In cases of myocarditis presenting with severe LV systolic dysfunction or cardiogenic shock, EMB should be considered [30].

The clinical course of myocarditis varies in each case depending on clinical presentation, aetiology, and disease stage. In approximately 50% of cases, acute myocarditis is self-limiting, with full resolution in 4 weeks. However, around 25% of individuals experience cardiac dysfunction, and 12–25% may rapidly worsen, potentially leading to death or progression to end-stage dilated cardiomyopathy, necessitating cardiac transplantation [37]. Factors associated with a poor prognosis include reduced LVEF, heart failure, New York Heart Association (NYHA) class II–IV, presence of LGE on CMR at baseline, and ventricular arrhythmia [38,39]. Early identification of these factors allows for the implementation of timely and appropriate therapeutic interventions to improve outcomes. Regular monitoring and follow-up are necessary to detect any progression or recurrence of myocarditis and allow for prompt adjustment of treatment strategies.

Of note, the presence of LGE was found to be the best independent predictor of all-cause and cardiac mortality [40]. In particular, LGE in the mid-wall and the septal locations was associated with a higher risk of major adverse cardiovascular events (MACE) [41,42].

## 4. Return-to-Play

The ESC guidelines and AHA/ACC scientific statement recommend exercise restriction post-myocarditis for a period of 3 to 6 months, depending on the clinical picture of the index event, which takes into account the duration of illness, LV systolic function, and the extent of LGE on CMR. After this period, the athlete should undergo reassessment, which should include 24-h Holter monitoring, echocardiogram, exercise ECG, and CMR [13,14]. The presence of complex ventricular arrhythmias, either on Holter monitoring and/or during exercise ECG, increases the risk of adverse outcomes post-myocarditis [14,43]. Both recommendations agree that the decision to RTP should be guided by normalised LVEF, normalised serum markers of myocardial injury and inflammation, and the absence of clinically relevant arrhythmias on 24-h Holter monitoring and exercise stress tests [13,14]. Furthermore, the EAPC recommends a periodical reassessment, especially during the first two years following the index event, due to the risk of recurrence and silent clinical progression [11]. For individuals recovering from COVID-19 myocarditis, the Canadian Working Group recommends a collaborative decision-making approach for evaluating their return to sports. Indeed, these athletes should understand the inherent limitations in myocarditis investigations, the potential implications of a myocarditis diagnosis, which commonly necessitates a sports restriction of 3 to 6 months or longer and the need for ongoing medical follow-up [44]. With regards to medication management, to date, there are no clinical trials conducted in athletes post-myocarditis. Treatment recommendations should be based on current guidelines in the general population [45]. Similarly, no particular sport has been associated with being higher risk when considering RTP for an athlete with myocarditis.

Despite myocarditis being a common disease phenomenon, several knowledge gaps exist, one of which includes the clinical significance of the persistence of LGE in those who remain asymptomatic post the index event. This is particularly the case with stable LGE at the follow-up CMR. The latest ESC guideline recommends the absence of myocardial inflammation and fibrosis on repeat CMR as guidance for RTP. The guidelines recommend repeat CMR in athletes with myocardial oedema or LGE on baseline CMR. In addition, the guidelines advise against moderate and high-intensity sports in individuals with extensive myocardial scar (>20% LGE) and persistent LV dysfunction. A shared decision-making approach should be adopted on a case-by-case basis in individuals with concerns during their evaluation [14]. Nonetheless, this is mainly based on expert opinion as shown in the level of recommendation Class IIa Level C [14]. The AHA/ACC consensus recognises this knowledge gap, stating that the role of myocarditis-related LGE resolution as a condition for an athlete’s return to competitive sports remains unresolved [13]. A guideline comparison summary for return-to-play following myocarditis is provided in Table 1.

A systematic review and meta-analysis of 11 studies found that the presence of LGE on baseline CMR was associated with an increased risk of adverse cardiac outcomes in patients with acute myocarditis. Furthermore, more extensive LGE, as well as the anteroseptal location of LGE, were more predictive of poor outcomes [46]. A study in athletes with ventricular arrhythmia showed that those with isolated non-ischaemic LGE may be associated with an increased risk of SCD or life-threatening arrhythmia [47]. Another study by Bohbot et al. examined dynamic changes in LGE in 204 patients with acute myocarditis who underwent follow-up CMR 3 to 12 months after diagnosis [48]. Findings revealed that a decrease in LGE of less than 50% or an increase in LGE were strong and independent predictors of major adverse cardiac events, emphasising the relevance of follow-up CMR for risk stratification. Furthermore, the persistence of LGE without myocardial oedema at a 6-month follow-up CMR was found to be associated with a worse cardiac prognosis [49].

A further knowledge gap relates to the 3- to 6-month period of exercise restriction following the index event. This recommendation is based on expert opinion, and to the best of our knowledge, there have been no studies comparing adverse outcomes after different sports restriction periods. In fact, a recent review article by Claessen et al. suggested a careful, individualised approach with a possible early RTP after a minimum of 4 weeks following symptom resolution [50].

Recently, there has been accumulated evidence relating to myocarditis actually presenting as the “hot phase” of arrhythmogenic cardiomyopathy (ACM) [51,52,53,54]. A systematic review by Monda et al. brought to light that 21.9% of complicated myocarditis (i.e., acute heart failure, reduced LVEF, or life-threatening ventricular arrhythmia) cases carry a genetic mutation related to cardiomyopathy [55]. This is particularly important for athletes who might carry a genetic mutation linked to ACM, as high-intensity exercise is discouraged in these cases (Class III, Level B) [14].

## 5. Shared Decision-Making Approach

An individualised and shared decision-making approach is a key element for RTP following myocarditis. The primary challenge in managing athletes with this condition lies in the scarcity of risk stratification protocols explicitly designed for them. Existing algorithms, derived from sedentary populations, do not account for the increased physical and metabolic demands athletes face, which can lead to fatal arrhythmias.

Considering the significant personal and financial implications of restriction from competitive sports, it is vital to involve athletes in the decision-making process [11]. They should be fully informed about their condition and the associated risks, allowing them to make educated decisions about their participation. With the athlete’s consent, physicians should respect these decisions and inform coaches and team physicians to ensure ongoing monitoring [11]. For RTP following acute myocarditis, decisions should consider cardiac function, myocardial scarring on CMR, and significant arrhythmias during exercise or Holter monitoring. Athletes who are asymptomatic and free of risk factors after four weeks may be considered for early RTP after a thorough evaluation, which includes CMR, exercise ECG, 24-h Holter testing, and serum cardiac markers [50]. A structured programme with gradual increases in intensity and ongoing clinical surveillance is recommended to monitor for new cardiovascular symptoms before full RTP [11,50].

Nonetheless, one must recognise that the approach to balance athlete autonomy with medical advice varies from country to country, and it is influenced by cultural norms, societal expectations, and local medical expertise. Furthermore, athlete’s autonomy may also be affected by legal systems across different countries. Therefore, shared decision-making may need to be adapted to align with the local medical and legal context [11]. In addition, the integration of multidisciplinary teams, including sports cardiologists, sports physicians, physiotherapists, and psychologists can provide comprehensive care tailored to each athlete’s needs to further improve the RTP decision-making process.

## 6. Clinical Recommendations and Future Research

**Figure 2:**
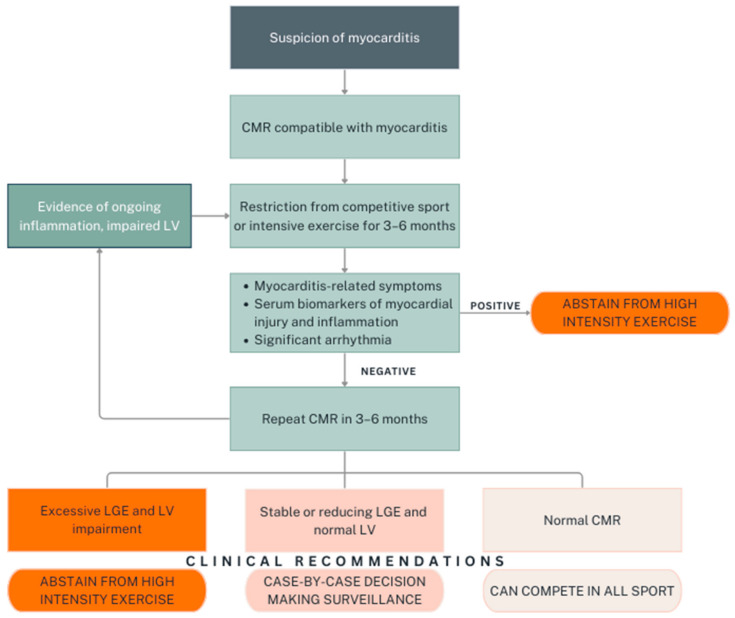
Proposed algorithm for return-to-play after myocarditis in athletes based on published guidelines.

Adapted from the latest ESC guidelines, follow-up CMR plays a key role in the decision-making process for RTP, together with other clinical parameters such as symptoms, serum biomarkers for myocardial injury and inflammation, as well as significant arrhythmias. A proposed algorithm for RTP in athletic individuals with myocarditis, which summarises the aforementioned guidelines with respect to RTP, is presented in Figure 2. In view of the high stakes involved in RTP decisions, particularly in elite athletes whose careers depend on their physical performance, the development of robust, evidence-based protocols is imperative. Longitudinal studies are warranted to track recovery trajectories and outcomes in athletes post-myocarditis.

While the ESC guideline underlines the importance of the role of CMR and LGE in monitoring myocarditis recovery in athletes, the presence of LGE at the time of repeat CMR needs further research. Prospective studies that allow for systematic data collection and follow-up can lead to evidence-based guidelines on this topic and offer a more detailed understanding of the disease course and outcomes in athletes. Ultimately, decisions about RTP must be tailored to individual athletes and should always incorporate a shared decision-making approach.

To the best of our knowledge, there have been no studies regarding the use of genetic analysis for athletes presenting with myocarditis. While this investigation can possibly reveal genetic findings suggestive of a future cardiomyopathy development, this needs to be carefully investigated and discussed with athletes since the long-term significance is uncertain.

## 7. Conclusions

Myocarditis poses a significant risk to athletes, particularly those engaged in high-intensity training. This condition not only affects physical health but also has psychological implications that require comprehensive monitoring. Early diagnosis and appropriate treatment are essential to prevent severe outcomes such as life-threatening arrhythmias and SCD. Current guidelines recommend a cautious approach to RTP, with thorough reassessment, including cardiac imaging and monitoring. A proposed algorithm for RTP after myocarditis in athletes, based on current guidelines, is presented. However, knowledge gaps remain, particularly regarding the persistence of myocardial scarring and the optimal duration of exercise restriction as well as the use of genetic analysis. Further research is warranted to refine these guidelines and improve risk stratification to ensure that athletes can safely resume their activities. A multidisciplinary approach, incorporating both medical and psychological support, is vital for the care of athletes recovering from myocarditis to better support RTP and overall well-being.

## Figures and Tables

**Table 1:** Guideline comparison for return-to-play following myocarditis.

Topic	AHA/ACC Scientific Statement (2015) [13]	EAPC Position Statement (2019) [11]	ESC Guidelines on Sports Cardiology (2021) [14]
Initial Evaluation	Resting echocardiogram, 24-h Holter, and an exercise ECG RTP in 3 to 6 months post-index event.	RTP in 3–6 months, depending on clinical severity.	Exercise stress testing, Holter monitoring, and imaging studies.
	(Class I; Level of Evidence C).	(Class IIb; Level of Evidence C).	(Class I; Level of Evidence B).
Risk Assessment Post-Recovery	Resuming training and competition is reasonable if all of the following criteria are satisfied: (a) Normalised ventricular function. (b) Normalised serum troponin and inflammatory markers. (c) No significant arrhythmias, such as frequent or complex ventricular or supraventricular ectopics, on Holter monitoring and graded exercise ECGs. Currently, there is no consensus on whether the resolution of myocarditis-related LGE should be a prerequisite for returning to competitive sports.	Resume training and competition if all of the following criteria are satisfied: (a) Normalised LV systolic function. (b) Normalised serum biomarkers of myocardial injury. (c) Absence of clinically significant arrhythmias, such as frequent or complex ventricular or supraventricular arrhythmias, on 24-h ECG monitoring and exercise testing.	RTP in 3–6 months can be considered if all of the following criteria are satisfied: (a) Symptom-free. (b) Normalised troponin and inflammatory biomarkers. (c) Normal LV systolic function on echocardiography and CMR. (d) Absence of ongoing inflammation or myocardial fibrosis on CMR. (e) Good functional capacity (f) No frequent or complex ventricular arrhythmias on Holter monitoring or exercise testing.
	(Class IIa; Level of Evidence C).	(Class IIa; Level of Evidence C).	(Class IIa; Level of Evidence C).

AHA/ACC = American Heart Association and the American College of Cardiology; CMR = cardiac magnetic resonance; EAPC = European Association of Preventive Cardiology; ECG = electrocardiogram; ESC = European Society of Cardiology; LGE = late-gadolinium enhancement; LV = left ventricle; RTP = return-to-play. Adapted from: Maron et al., 2015; Pelliccia et al., 2019; Pelliccia et al., 2021 [11,13,14].

## Data Availability

No new data were created or analyzed in this study.
